# Supplementary material for: Highly Fluorescent Green Carbon Dots as a Fluorescent Probe for Detecting Mineral Water pH
Source: Sensors (Basel). 2019 Sep 3;19(17):3801. doi: 10.3390/s19173801 (PMC6749429; doi:10.3390/s19173801)
Supplement: Supplementary file 1 [file sensors-19-03801-s001.pdf]

Supplementary Information

Highly fluorescent green carbon dots as a fluorescent probe for detecting mineral water pH

Tingyu Wang [1,2], Lei Li [1,2], Yamin Wu[1,2] and Guoqing Chen [1,2,*]

[1] School of science, Jiangnan University, Wuxi, 214122, P. R. China; jncgq@jiangnan.edu.cn
[2] School of Science, Jiangsu Provincial Research Center of Light Industrial Optoelectronic Engineering and Technology, Wuxi, 214122, China;; cjq2098@163.com
* Correspondence: jncgq@jiangnan.edu.cn; Tel.: +86-13906176695

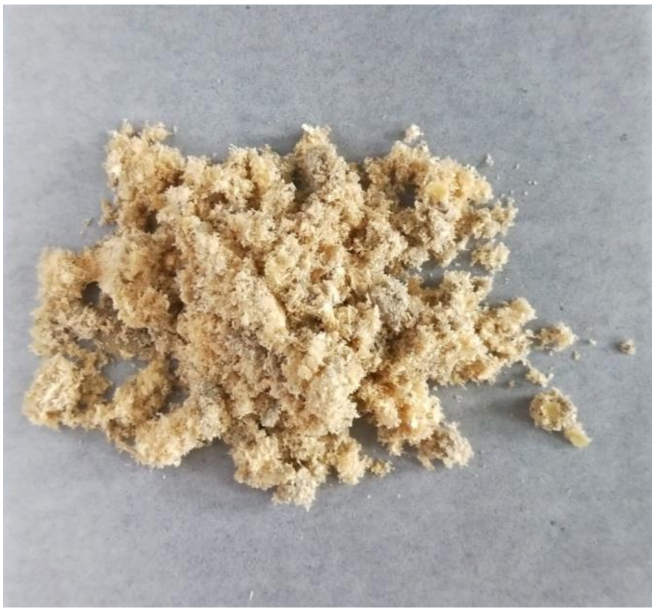

Figure S1. The image of the CDs solid powder

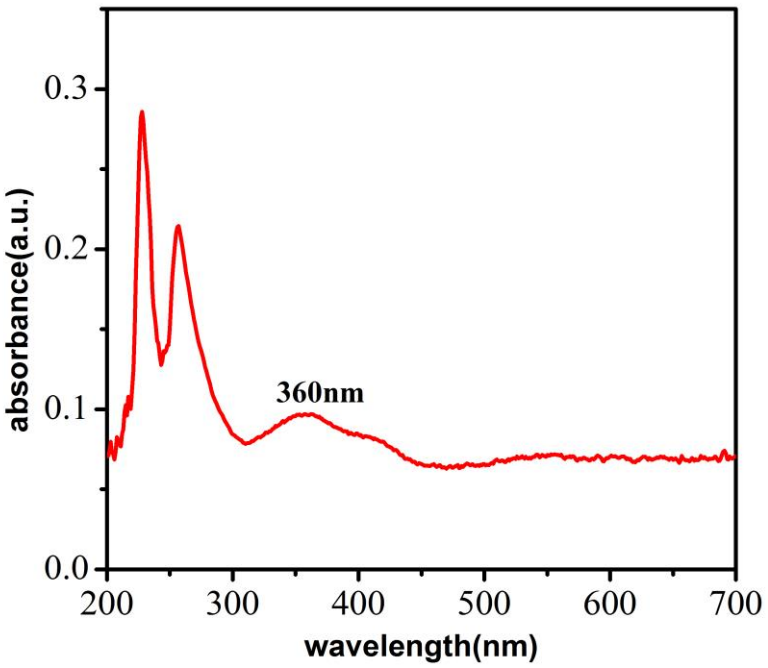

Figure S2. Absorption spectrum of the CDs.

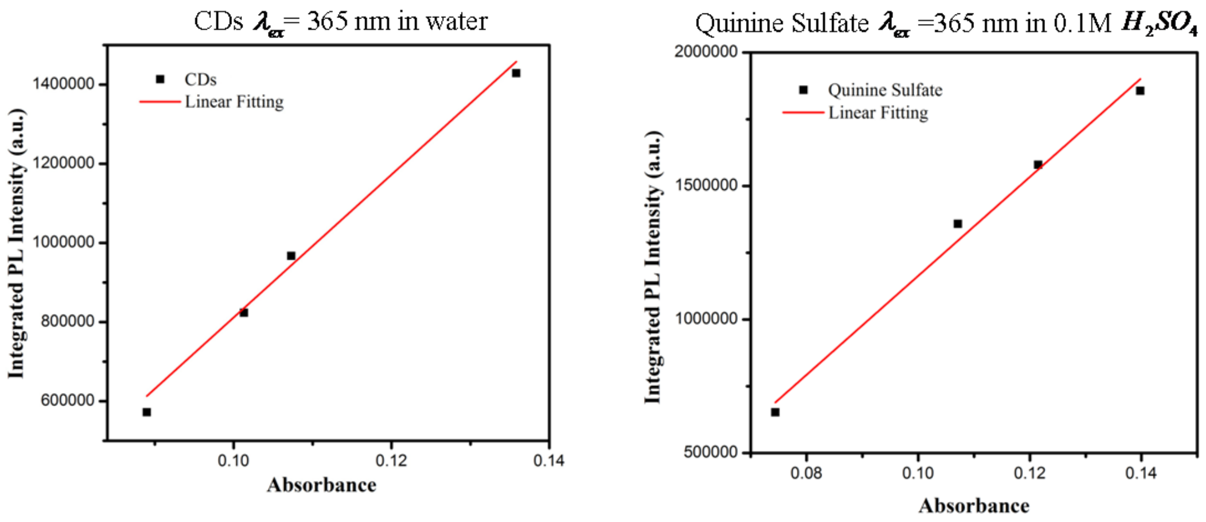

	CDs				Quinine Sulfate			
Absorbance	0.089	0.1013	0.1073	0.1358	0.0744	0.1071	0.1215	0.1398
Integrated PL	572100	823200	966900	1429000	652300	1358000	1579000	1856000
Slope	1.81×10^{6}				1.85×10^{6}			
QY	53.81%				55%			

Figure S3. Plot of integrated PL intensity of the CDs and quinine sulfate (referenced dye) as a function of optical absorbance at 365 nm and relevant data.

.

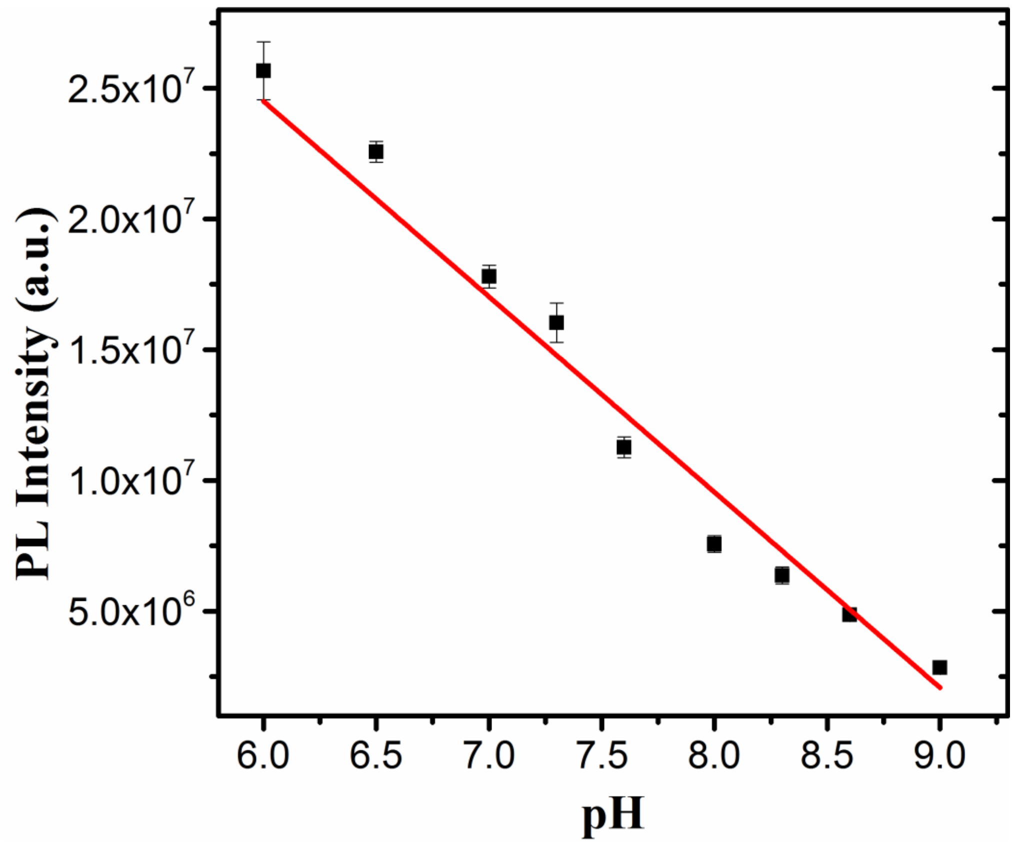

Figure S4. A linear relationship between PL intensity and pH (from6.0 to 9.0).

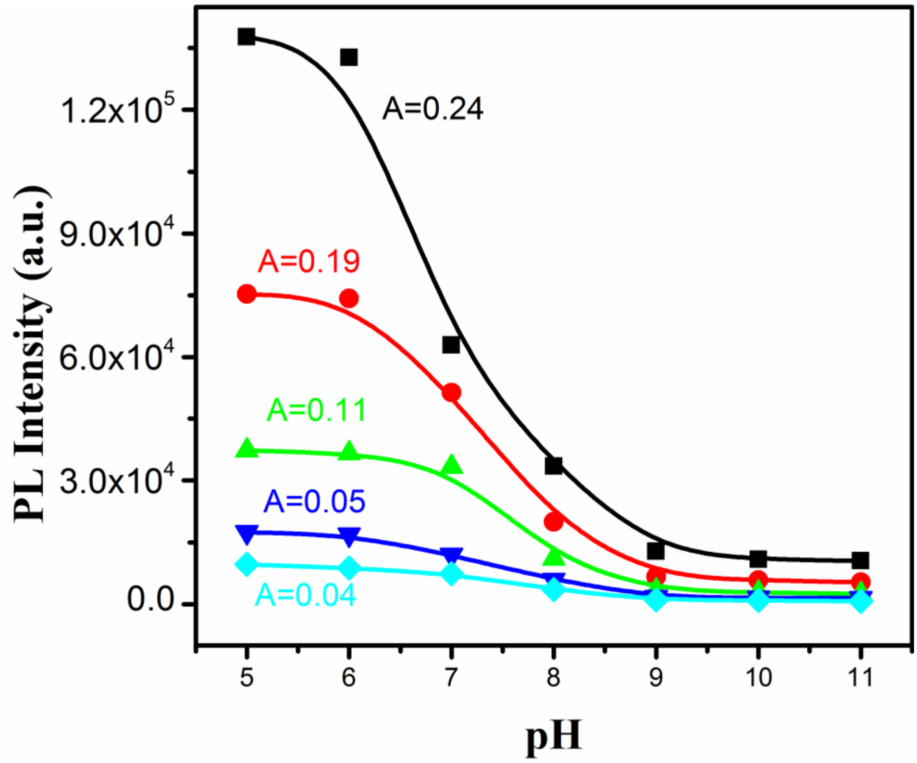

Figure S5. Response of CDs to pH at different absorbances (at 390 nm).

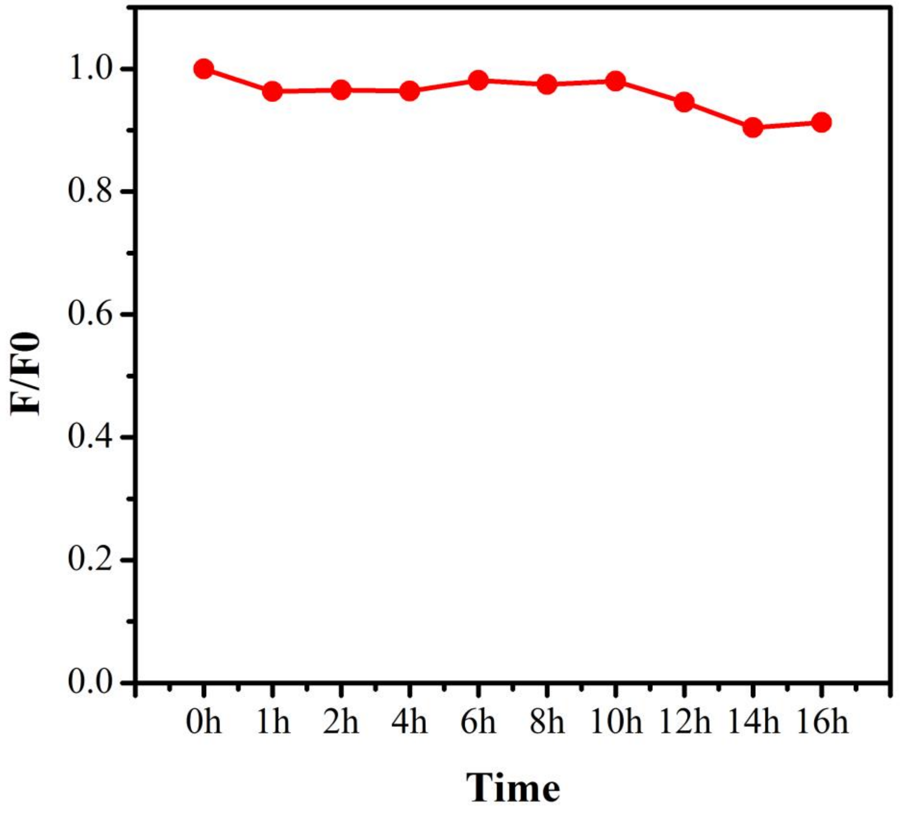

Figure S6. Photo stability of CDs under the irradiation of a 600W light at various times.
